# Molecular Characterization of a Date Palm Vascular Highway 1-Interacting Kinase (*PdVIK*) under Abiotic Stresses

**DOI:** 10.3390/genes11050568

**Published:** 2020-05-19

**Authors:** Ibtisam Al-Harrasi, Himanshu V. Patankar, Rashid Al-Yahyai, Ramanjulu Sunkar, Pannaga Krishnamurthy, Prakash P. Kumar, Mahmoud W. Yaish

**Affiliations:** 1 Department of Biology, College of Sciences, Sultan Qaboos University, P.O. Box 36, Muscat 123, Oman; i.alharrasi@gmail.com (I.A.-H.); himanshu30@gmail.com (H.V.P.); 2 Department of Crop Sciences, College of Agricultural and Marine Sciences, Sultan Qaboos University, P.O. Box 34, Muscat 123, Oman; alyahyai@squ.edu.om; 3 Department of Biochemistry and Molecular Biology, Oklahoma State University, Stillwater, OK 74078, USA; ramanjulu.sunkar@okstate.edu; 4 Department of Biological Sciences, Faculty of Science, and NERI, National University of Singapore, Singapore 117543, Singapore; eripk@nus.edu.sg (P.K.); prakash.kumar@nus.edu.sg (P.P.K.)

**Keywords:** vascular highway 1-interacting kinase, date palm, abiotic stress, drought, salinity, phosphorylation

## Abstract

The date palm (*Khalas*) is an extremophile plant that can adapt to various abiotic stresses including drought and salinity. Salinity tolerance is a complex trait controlled by numerous genes. Identification and functional characterization of salt-responsive genes from the date palm is fundamental to understand salinity tolerance at the molecular level in this plant species. In this study, a salt-inducible vascular highway 1-interacting kinase (*PdVIK*) that is a MAP kinase kinase kinase (MAPKKK) gene from the date palm, was functionally characterized using in vitro and in vivo strategies. *PdVIK*, one of the 597 kinases encoded by the date palm genome possesses an ankyrin repeat domain and a kinase domain. The recombinant PdVIK protein exhibited phosphotyrosine activity against myelin basic protein (MBP) substrate. Overexpression of *PdVIK* in yeast significantly improved its tolerance to salinity, LiCl, and oxidative stresses. Transgenic Arabidopsis seedlings overexpressing *PdVIK* displayed improved tolerance to salinity, osmotic, and oxidative stresses as assessed by root growth assay. The transgenic lines grown in the soil also displayed modulated salt response, compared to wild-type controls as evaluated by the overall plant growth and proline levels. Likewise, the transgenic lines exhibited drought tolerance by maintaining better relative water content (RWC) compared to non-transgenic control plants. Collectively, these results implicate the involvement of *PdVIK* in modulating the abiotic stress response of the date palm.

## 1. Introduction

Plant growth and development are directly affected by environmental stresses such as salinity, drought and extreme temperature [1]. Soil salinization causes considerable loss of agricultural land, affecting crop production, particularly in arid and semiarid regions [2,3,4]. To cope with salt stress, plants have evolved several mechanisms including restricted Na^+^ accumulation in sensitive tissues, sequestration of Na^+^ in the vacuoles, maintenance of K^+^ homeostasis, accumulation of compatible solutes (e.g., sugars, amino acids, and glycine betaine) and late embryogenesis abundant (LEA) proteins, and elevated levels of enzymatic and non-enzymatic antioxidants [5,6,7,8,9]. Therefore, diverse classes of genes coding for transporters, enzymes, structural components, as well as regulatory proteins such as transcription factors, phosphatases, and protein kinases participate in this complex process [5,10].

Protein kinases are part of the numerous signal transduction pathways participating in the phosphorylation of specific target proteins [11]. The phosphorylated target proteins can undergo several fates, including modulation of protein activity, cellular localization, and association with other proteins [12]. On the basis of substrate specificity of the kinase domains, three major classes of kinases are identified: tyrosine kinases (TK), serine/threonine kinases (STK), and histidine kinases (HK) [13]. STK and HK are the most common types of protein kinases found in plants [14]. In addition to their involvement in various growth and developmental processes, protein kinases play a key role in regulating biotic and abiotic stress responses [11,15]. For example, salt overly sensitive-2 (SOS2), a serine/threonine-protein kinase, is responsible for phosphorylating plasma membrane and tonoplast Na^+^/H^+^ antiporters, which promotes Na^+^ extrusion and vacuolar compartmentalization, respectively [16,17]. Additional regulators of serine/threonine kinases include vacuolar H^+^-ATPase, Cl^-^ channels [18]. A previous study has shown that constitutive expression of a maize calcium-dependent protein kinase (*ZmCPK11*) enhanced salt tolerance in Arabidopsis by regulating Na^+^ and K^+^ homeostasis, maintaining photosystem II, upregulating various transcription factors, as well as transporters (SOS1, NHX1, and HKT1) under salt stress [19].

The date palm (*Phoenix dactylifera* L.) is one of the first cultivated trees on earth that may possess a unique well-evolved salt tolerance mechanism [20,21], therefore, identifying genes and deciphering salinity tolerance mechanisms in this plant species is critical. In a number of diverse plant species, the transcript abundance of a variety of kinases was shown to be altered in response to salt stress [22,23,24]. Furthermore, their functions have also been studied using transgenic approaches, mainly in Arabidopsis [19,22,25,26,27]. Previously, we reported on the identification of a MAP kinase kinase kinase (MAPKKK)—named as serine/threonine kinase (STK)—that is expressed in response to salt stress in the date palm [28]. At the protein level, it has 85% identity with the Arabidopsis VIK (VH1-interacting kinase—At1g14000), which is related to the MAPKKK family and plays a role in responses to abiotic stress conditions, and it belongs to Raf-like MAPKKK, categorized as part of the C1 subgroup, and acts as a downstream adaptor protein for BRL2/VH1 (BRL2 receptor-like kinase) [29]. Therefore, it exhibits a vital role in the final stage of vascular development in Arabidopsis. The interaction between VIK and BRL2/VH1 is also involved in auxin and brassinosteroid signaling [30]. The VIK of date palm (*PdVIK*) was induced in both leaves and roots under high salinity and has also been shown to be associated with salt tolerance in a wild-type yeast strain [28]. 

Several salt-induced kinases have been identified in the date palm [31]. However, no reports are available on the functional characterization of stress-induced kinases in this plant species. In the present study, the importance of the *PdVIK* gene in abiotic stress tolerance was evaluated both in yeast and Arabidopsis. The results revealed that the overexpression of *PdVIK* in yeast enhances its tolerance to salinity, oxidative, and ionic stresses. Similarly, transgenic Arabidopsis seedlings overexpressing *PdVIK* were more tolerant to salt and drought stresses.

## 2. Materials and Methods

### 2.1. In Silico Protein Sequence Analysis

The amino acid sequences of the protein kinase family of the date palm, rice, and Arabidopsis were retrieved from the National Center of Biotechnology Information (NCBI) database. The sequences were aligned by ClustalW software [32], using the default parameter incorporated within MEGA 7 program [33]. The resulting alignments were used in the construction of phylogenetic trees using the Neighbor-Joining (N-J) algorithm with a bootstrap analysis of 1000 replicates. The conserved motifs, within the protein kinase family of the date palm, were identified using the multiple expectation maximization for motif elicitation (MEME) platform (http://alternate.meme-suite.org/). MEME parameters were set to detect a maximum of 40 motifs, with a coverage width of 6 to 50 amino acid residues [34]. PROSITE databases were used to obtain the conserved domains among the protein sequence [35]. Hydrophobicity using Kyte–Doolittle scale profile of the PdVIK protein, theoretical isoelectric point (pI), and molecular weight (Mw) of the target protein was predicted through ExPASy (ProtScale) tool [36,37]. 

### 2.2. In-Silico Analysis of the Putative Promoter Sequences of the Kinase Genes

The putative promoter sequences of 53 abiotic stress-responsive kinases of the date palm were retrieved from NCBI database. The length of promoter sequences varied between 366 and 2000 bp, due to long gaps in the genome draft. The sequences were further submitted to the PlantPAN 3.0 (http://plantpan.itps.ncku.edu.tw/promoter.php) database to identify putative regulatory elements and their recognition sites. The distributions of regulatory elements that are known to be involved in abiotic stress compared with other general regulatory elements are represented in a pie diagram. The Plant CARE (cis-acting regulatory elements) online database available at http://bioinformatics.psb. ugent.be/webtools/plantcare/html/ [38], was also used for the same purpose.

### 2.3. Production of Recombinant PdVIK Protein in E. coli

The full-length cDNA of *PdVIK* was amplified using primers containing *Nco*I and *Bam*HI sites (Supplementary Table S1), and the amplicon was cloned into a pTYB21 vector (New England Biolabs, Ipswich, MA, USA), where its N-terminus was fused in-frame with the Intein tag containing the chitin-binding domain. The ligated plasmid was introduced by electroporation into *E. coli* DH10B cells for amplification. Subsequently, the sequence confirmed construct was introduced into an *E. coli* ER2566 strain for protein expression. The PdVIK was expressed under the control of the *Lac* operon. The recombinant PdVIK protein was purified using Intein Mediated Purification with an Affinity Chitin-binding Tag (IMPACT) system (New England Biolabs, Ipswich, MA, USA), following the manufacturer’s instructions with minor modifications. Protein production was induced using 0.5 mM isopropyl β-D-1-thiogalactopyranoside (IPTG) at 18–20 °C for 6 hours. After centrifugation at 10,000 g, the cells were harvested and suspended in column buffer (20 mM Tris-HCl, 500 mM NaCl, 1 mM EDTA, and 0.1% Triton X-100, pH 6.8) and cells were lysed by sonication. The PdVIK protein was eluted using a column buffer supplemented with 50 mM of dithiothreitol (DTT). The protein was further purified from salts and concentrated using a centrifugal filter unit with a molecular cut-off of 10 kD (Merck Millipore, Billerica, MA, USA).

### 2.4. Enzymatic Phosphorylation Activity Assay of PdVIK

The activity of the purified recombinant PdVIK protein was evaluated in a phosphorylation activity assay. The enzymatic reaction included kinase buffer (catalog number PV3189, Sigma, USA), 8 µg myelin basic protein substrate (Thermo Scientific, catalog number M1891, USA), 0.125 mM ATP (Sigma, St. Louis, MO, USA), and either 0.72 µg or 1.44 µg, of the purified PdVIK protein. Two negative controls were also used (substrate and enzyme were excluded). The reactions were incubated at room temperature for 1 h. Each reaction mixture (20 µL) was fractionated in 15% SDS-PAGE, then blotted onto a 0.45 µm Immobilon-FL PVDF membrane using the semi-dry protein transfer method. A standard Western blot procedure was carried out using an anti-mouse phospho-tyrosine antibody (Cell Signaling Technology, Beverly, MA, USA, Catalogue number 9411), at 1:1000 dilution. The HRP-linked goat anti-mouse secondary antibody (Abcam, UK, catalog number ab205719) was used at 1:1000 dilution. The signals of the Western blot were detected using Clarity ECL (Bio-Rad, Hercules, CA, USA) and visualized using the ChemiDoc™ Touch Imaging System (Bio-Rad, Hercules, CA, USA). The image J software [39], was used to digitally quantify the phosphorylated bands on the membrane.

### 2.5. Molecular Cloning and Heterologous Expression of PdVIK in Yeast

*PdVIK* cDNA was obtained from a cDNA library prepared from salt-treated date palm roots [28]. *Saccharomyces cerevisiae* strains *INVScI* (wild-type) and *BYT458* (BY4741; ena1-5Δ::loxP nha1Δ::loxP vnx1Δ::loxP) [40], a mutant strain kindly donated by Professor Hana Sychrova, the Czech Republic, were used for the complementation assay. The *PdVIK* cDNA was cloned into the yeast expression vector pYES-DEST52, downstream of a galactose-inducible GAL1 promotor using a gateway cloning strategy (Invitrogen, Carlsbad, CA, USA). The resultant recombinant vector was mobilized into the *E. coli* DH10B strain using a standard electroporation technique. The recombinant plasmid (PdVIK), as well as an empty vector (EV) were introduced into the yeast cells using a PEG-lithium acetate method with Yeastmaker^TM^ yeast transformation system 2 (Clonetech laboratories, USA) following the manufacturer’s instructions. Subsequently, the transformed yeast cells were selected based on auxotrophic selection marker gene *URA3* on solid synthetic medium (SSM) lacking uracil: 0.67% yeast nitrogen base, 1 mM potassium chloride, 0.6% Sucrose, 2% galactose, 10% amino acid stock (0.02% L-Histidine, 0.06% L-Leucine and 0.02% L-Methionine) and 2% Difco nutrient-free agar. Three independent transgenic cell lines (colonies) were used in the subsequent analysis. 

### 2.6. Functional Yeast Spot Assay

The yeast cells were tested for their stress tolerance using the spot assay. In this assay, the yeast cells were cultured initially in liquid synthetic medium (LSM) (0.67% yeast nitrogen base, 1 mM potassium chloride, 0.6% Sucrose, 2% dextrose and 10% amino acid stock (0.02% L-Histidine, 0.06% L-Leucine and 0.02% L-Methionine)) overnight at 30 °C, and shaken at 200 rpm. Precultured yeast cells were pelleted and washed twice with sterile distilled water, and the optical density at 600 (OD_600_) was adjusted to 1.0. Five serial dilutions (10^−1^, 10^−2^, 10^−3^, 10^−4^, and 10^−5^) were prepared from the stock in LSM. A volume of 10 μL of each dilution was spotted on SSM supplemented with 2% galactose (control plates), or with 300 mM NaCl (salt stress), 3 mM H_2_O_2_ (oxidative stress), as well as 10 mM LiCl, and 500 mM KCl (cation stress). EV or PdVIK cells were spotted on each plate in order to compare the behavior under control and abiotic stress conditions. The plates were incubated at 30°C for 3-5 days, and their growth rates were recorded. 

### 2.7. Cation Sensitivity Assay

Three replicates each of the EV, and PdVIK yeast cells were grown on LSM supplemented with 2% dextrose and maintained in a shaker (30 °C with 200 rpm) for two days. The yeast growth assay was performed on LSM with 2% galactose only (control) or LSM supplemented with 100 mM or 200 mM KCl. The OD_600_ of each culture was adjusted to 0.05, and the yeast growth (OD_600_) was measured over a period of three days.

### 2.8. Intracellular Measurement of Na^+^ and K^+^ Ions in Yeast Cells

Yeast strains were grown in 10 mL of LSM, either plain or supplemented with 25 mM NaCl. Briefly, cells at an OD_600_ of 0.3–0.4 were harvested by centrifugation, washed twice with deionized water, and resuspended in 1 mL water. The cells were boiled for 20–30 min, and the supernatant of the boiled cells was diluted 10-fold, and Na^+^ and K^+^ concentration were measured using a flame photometer (Systronics, Ahmedabad, India). The Na^+^ and K^+^ concentrations were expressed in µmol/1.0 × 10^6^ cells, as previously described [41]. 

### 2.9. Cloning and Heterologous Expression of PdVIK in Arabidopsis

The *PdVIK* cDNA was cloned into the plant binary vector, pEarleyGate 203 (TAIR stock Id- CD3-689), in-frame with the epitope Myc-tag, under the control of the cauliflower mosaic virus *CaMV 35S* promoter using the gateway cloning technology (Invitrogen, USA). The resultant recombinant vector (EarleyGate 203::*PdVIK*) was introduced into *Agrobacterium tumefaciens* LBA4404 strain (Invitrogen, USA) by electroporation. Transgenic colonies were confirmed using PCR with gene-specific primers (Supplementary Table S1). *PdVIK* was used to genetically transform *Arabidopsis thaliana* L. (ecotype Columbia Col-0) via the floral dip method [42]. After maturation, the seeds were collected and sown on a potting mixture, and the transgenic Arabidopsis T0 plants were selected by spraying with 0.01% glufosinate-ammonium solution (BASTA^®^) (Bayer, Germany). The selected plants were further confirmed by PCR using the 35S forward promoter and gene-specific reverse primers (VIKR), as well as gene-specific forward (VIKF) and OCS terminator reverse primers (Supplementary Table S1). Seeds were collected from the T0 plants, grown on MS medium plates supplemented with BASTA^®^, and transgenic lines with single insert were selected based on the Mendelian genetic segregation ratio (3:1). The plants were grown for two more generations, and independent homozygous lines were obtained (T3).

### 2.10. Stress Tolerance Analysis of PdVIK Transgenic Arabidopsis Lines on MS Medium

Abiotic stress tolerance analysis was conducted using wild-type (WT) and two independent homozygous transgenic lines (TL1 and TL2). Seeds from each line were cold-stratified prior to germination on half-strength Murashige and Skoog (MS) agar medium for four days. Subsequently, the seedlings were transferred to MS agar containing 150 mM NaCl, 200 mM mannitol, and 3 mM H_2_O_2_. After two weeks of growth, the root lengths of the WT and transgenic lines were measured. This experiment was conducted using four technical replicates.

### 2.11. Stress Tolerance Analysis of Soil-Grown PdVIK Transgenic Lines 

Transgenic and WT seeds were germinated on 0.5 strength MS medium for four days. Then, plantlets were transferred to pots (half-liter) containing potting mixture. The pots were transferred to a growth chamber maintained at 22 °C, 60% relative humidity, and 16 h light/8 h dark cycle. All the pots were watered to field capacity with distilled water for three consecutive weeks. Thereafter, plants were either watered with distilled water (control), or with 200 mM NaCl solution every four days for 14 consecutive days (salinity treatment), or left without irrigation for two weeks (drought treatment). The experiment was conducted in three biological replicates, with four technical replicates in each treatment group. Soil electrical conductivity (EC) for three groups was measured using an Em50 Digital Data Logger (Decagon Devices, WA, USA).

At the end of the stress treatment, the total chlorophyll [43], Relative Water Content (RWC) [44], and proline [45] content were determined. Plant recovery experiments after drought treatment were carried out as previously described [9,46]. 

### 2.12. Estimation of Na^+^ and K^+^ Levels in Arabidopsis Plants

Arabidopsis seedlings were dried at 70 °C for 24 h, and were used for Na^+^ and K^+^ estimations. In brief, Na^+^ and K^+^ were extracted from plant samples by 0.5 M nitric acid and maintained in a shaker (100 rpm) for 2 days at room temperature. Subsequently, the concentrations of the solubilized Na^+^ and K^+^ in the filtered extract were measured using a Systronics flame photometer 128 (Systronics, India). Na^+^ and K^+^ standards were used to quantify the ion concentrations in the samples. The flame photometer readings were converted to micrograms of Na^+^ and K^+^ per milliliter of extract, and finally, the concentrations were expressed as micromolar per gram of dry weight, as described earlier [47]. 

### 2.13. Protein Extraction from Plants and Western Blotting

To confirm the expression level of PdVIK in Arabidopsis transgenic lines, total proteins were isolated from three-week-old Arabidopsis seedlings and used for Western blot analysis. Briefly, the plant samples were ground into a fine powder using extraction buffer (100 mM Tris-HCl, pH 6.8, 100 mM NaCl, 1mM phenylmethylsulfonyl fluoride (PMSF), and 5% glycerol). The total protein lysate was resolved in a 12% TGX Stain-Free™ FastCast™ Acrylamide gel (Bio-Rad, USA). Subsequently, the proteins were transferred to a 0.45 µm Immobilon-FL PVDF membrane (Merck Millipore, USA) using a Trans-Blot® Turbo™ Transfer System (Bio-Rad, USA). The anti-Myc-tag primary antibody (9E10) (Abcam, UK, catalog number ab117499) was used at 1:2,500 dilution and the HRP-linked Goat Anti-Mouse secondary antibody (Abcam, UK, catalog number ab205719) was used at 1:1000 dilution and the immunoblots were detected, as mentioned earlier in this study.

### 2.14. Statistical Analysis

One-way analysis of variance (ANOVA) was used to compare the difference between the mean of tested variables. Duncan’s Multiple Range Test (DMRT) was used to measure the significance of the tested variables at *p* < 0.05.

## 3. Results

### 3.1. Date Palm Genome Encodes Ten Families of Protein Kinase Genes

**Figure 1:**
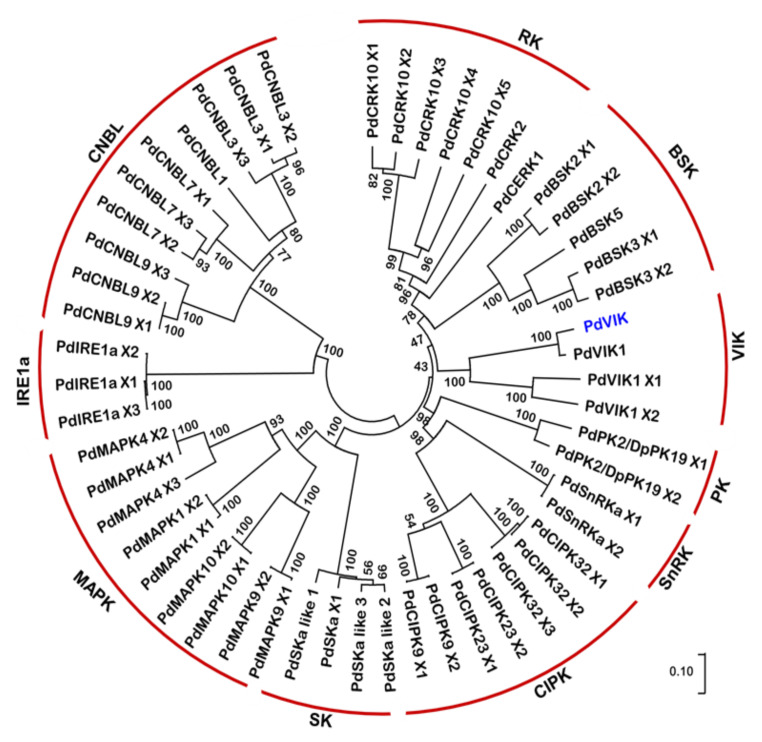
Phylogenetic analysis of 53 date palm protein kinases related to the abiotic stress response. The constructed neighbor-joining tree showed that the date palm protein kinases could be divided into ten different groups, shown bracketed in red lines. The values on the nodes represent the percentage of 1000 replicates in a bootstrap. There are four isoforms of date palm vascular highway 1-interacting kinases (VIK) in the database, PdVIK (blue font) is the candidate protein selected for further analysis in this study.

Initially, an in silico analysis was conducted using the available information in GenBank to identify the entire protein kinase gene family in the date palm. The analysis yielded a total of 597 kinases (Supplementary Table S2). From these, the kinases associated with abiotic stress responses in date palm were filtered based on their similarities to the stress-associated kinases of different plant species [48]. This analysis identified 53 genes as abiotic stress-responsive kinases in the date palm (Supplementary Table S3). Phylogenetic analysis grouped these kinases into ten different classes (Figure 1): the receptor-type protein kinases (RK), including Chitin elicitor receptor protein kinases (CERK) and cysteine-rich receptor-like protein kinases (CRK); the serine/threonine-protein kinases, BSK and PK; the vascular highway 1-interacting kinases (VIK) (MAPK group, 2002); the SNF1-related protein kinases (SnRK); the CBL-interacting serine/threonine-protein kinases (CIPK); the shaggy-related protein kinases (SK); the mitogen-activated protein kinases (MAPK); the endoribonuclease (IRE1a); and the calcineurin B-like protein (CNBL).

The phylogenetic analysis revealed a high similarity between most of the date palm kinases and their orthologous genes from Arabidopsis and rice (Figure S1). VIK belongs to Raf-like MAPKKK belonging to the C1 subgroup (MAPK group, 2002). *PdVIK* (Accession number XM_008811351.3) transcript was one of the genes upregulated in both the leaf and root tissues of date palms exposed to salinity [28]; therefore, it was chosen for functional analysis in this study. 

The PdVIK protein has 458 amino acids (47.3 kDa) with an average isoelectric point (pI) of 8.64 and this protein displayed 85% sequence identity with Arabidopsis At1g14000, a Vascular Highway 1 interacting kinase-VIK [30]. 

As revealed by the multiple sequence alignment, the PdVIK protein shares conserved domains with the VIKs from Arabidopsis, potato, and rice (Figure S2). The C1 MAPKKKs (VIKs) code for N-terminal ankyrin repeat domains, and are mostly annotated as integrin-linked (ILKs) proteins with mostly unknown function (MAPK group, 2002) [49]. Analysis of the structural motifs of PdVIK revealed that it shares seven motifs with PdILK and PdPK2/PdPK19 kinases (Figure S3). However, a unique motif is only shared between PdVIK and PdILK1 (asterisk), suggesting that this motif may have an essential role in these two proteins.

**Figure 2:**
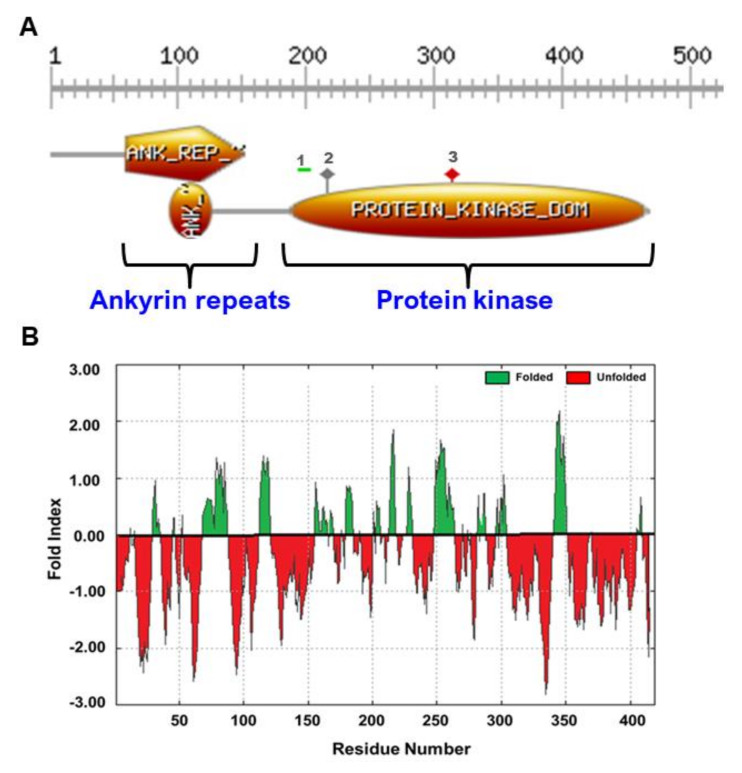
The conserved domains and the hydrophobicity profile of PdVIK. The PROSITE scan shows that PdVIK has two distinct domains: ankyrin (ANK) and protein kinase catalytic (PKc) domains (**A**). The PKc domain consists of polypeptide substrate binding site (1), ATP binding site (2), and protein kinase active site (3), grayscale on top of the domains indicates the number of amino acid sequence (domain ranges). The hydrophobicity profile of PdVIK protein plotted, according to the Kyte–Doolittle scale, shows the presence of the large hydrophilic regions, indicated in red (**B**).

In silico analysis of the PdVIK protein, using PROSITE databases revealed the presence of ankyrin (ANK) repeats and protein kinase catalytic (PKc) domains (Figure 2A). PKc contained the polypeptide substrate binding site, the ATP binding site, and the protein kinases active site (ACT site). The active-site of PdVIK includes a highly conserved aspartic acid residue (D), which is known to enhance the enzyme activity [50]. The computational analysis of the PdVIK protein hydrophobicity profile predicted the presence of hydrophilic regions (Figure 2B). 

Promoter analysis, by searching against the PlantPAN 3.0 (http://plantpan.itps.ncku.edu.tw/promoter.php) and Plant CARE databases [38], revealed the presence of 40% of abiotic stress-responsive elements in the *PdVIK* putative promoter region (2000 bp). The most abundantly abiotic stress-responsive elements recognized among this region were AP2/ERF, WRKY, bZIP, MYC, bHLH, trihelix, NAC, MYB, ZF-HD, GT1, DRE and CAAT (Figure S4). 

### 3.2. PdVIK Exhibited Tyrosine Phosphorylation Activity

Based on in silico analysis and the amino acid sequence annotation, PdVIK is a kinase that catalyzes the transfer of phosphate groups from ATP to a protein substrate. 

**Figure 3:**
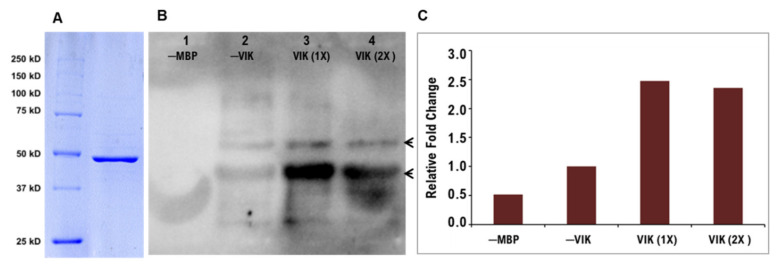
Determination of kinase activity of the recombinant PdVIK protein. The purified PdVIK protein (Molecular weight ~47.3 kDa) shown in the SDS-PAGE image (**A**). Immunoblot image shows in vitro phosphorylation activity of PdVIK at tyrosine residues of the myelin basic protein (MBP) substrate (**B**). Four reaction mixtures (20 µL) were loaded as follows: lane 1 includes kinase buffer, ATP, and purified PdVIK enzyme, without the MBP substrate (─MBP); lane 2 consists of the MBP substrate but without the PdVIK enzyme (─VIK); the last two lanes include the complete reaction mixture but with different amounts of the protein, namely, 0.72 μg (1 × VIK) and 1.44 µg (2 × VIK). Tyrosine phosphorylation was detected using anti-mouse phospho-tyrosine primary antibody (1:1000 dilution) and HRP-linked goat anti-mouse secondary antibody (1:1000 dilution), arrows indicate two phosphorylated bands of MBP (molecular weight ~18 kDa and ~22 kDa). The bar graph represents the relative fold change of phosphorylated band intensity on the immunoblot when compared to the reaction without PdVIK enzyme (─VIK) (**C**).

The purified PdVIK was obtained through Intein tag-mediated fusion, at its N-terminus. The phosphorylation activity of the purified recombinant PdVIK protein (Figure 3A) was assessed in vitro against the myelin basic protein (MBP) substrate. An evident tyrosine phosphorylation activity was detected in the immunoblot assay using the specific antibodies (Figure 3B). Quantification of this activity revealed that the tyrosine phosphorylation of MBP was significantly increased (2.5-fold) in the presence of PdVIK, compared with the negative control (─VIK) (Figure 3C). It is clear that no phosphorylation signals were detected in the absence of MBP (lane 1), so none of the tyrosine residues of PdVIK enzyme were phosphorylated by bacterial kinases during the expression and the detectable signals in lane 3 and 4 resulted from the PdVIK phosphorylation of tyrosine residues on the substrate (MBP). Therefore, phosphatase treatment of PdVIK was not included in this experiment.

### 3.3. Expression of PdVIK in Yeast Enhanced Growth Under Abiotic Stress Conditions 

**Figure 4:**
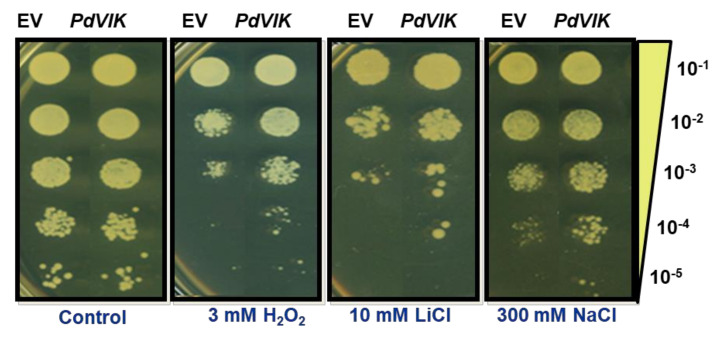
Overexpression of *PdVIK* in a salt-sensitive yeast strain (BYT458). The Transgenic *PdVIK* and the empty vector (EV) yeast cells spotted on solid synthetic medium (SSM) (control) and SSM supplemented with 3 mM H_2_O_2_ (oxidative stress), 10 mM LiCl (ionic stress), and 300 mM NaCl (salt stress) to assay tolerance potential of the transgenic yeast. The experiment was conducted three times and the EV was used as a negative control in the experiment.

The *PdVIK* gene was cloned into a yeast vector controlled by a GAL1 inducible promoter and overexpressed in yeast cells to assess the ability of the gene to confer salt tolerance. Our previous work showed that WT *S. cerevisiae* could withstand high NaCl concentrations [28]. Hence a mutant yeast strain (BYT458) with impaired salt tolerance was used to assay the function of *PdVIK*. The growth response of the yeast was tested on SSM supplemented with 300 mM NaCl or 10 mM LiCl or 3 mM H_2_O_2_, representing the salinity, ionic, and oxidative stresses, respectively. Under control conditions, both empty vector (EV) and PdVIK cells had similar growth rates. However, transgenic yeast cells showed improved NaCl tolerance compared to EV-transformed control cells (Figure 4). Similarly, under H_2_O_2_ and LiCl stresses, the PdVIK-transformed yeast could grow beyond the fourth dilution level and displayed improved tolerance compared to the non-transformant control (Figure 4). 

The accumulation of Na^+^ and K^+^ plays a significant role in salt stress tolerance. To determine whether or not the intracellular Na^+^ and K^+^ concentrations differ between EV and transgenic yeast cells (which could, at least partly account for salt tolerance or sensitivity), the ion levels were measured in yeast cells grown in LSM supplemented with 0 mM or 25 mM NaCl. The salinity level (25 mM), was selected because it is the highest non-lethal concentration for yeast cells.

**Figure 5:**
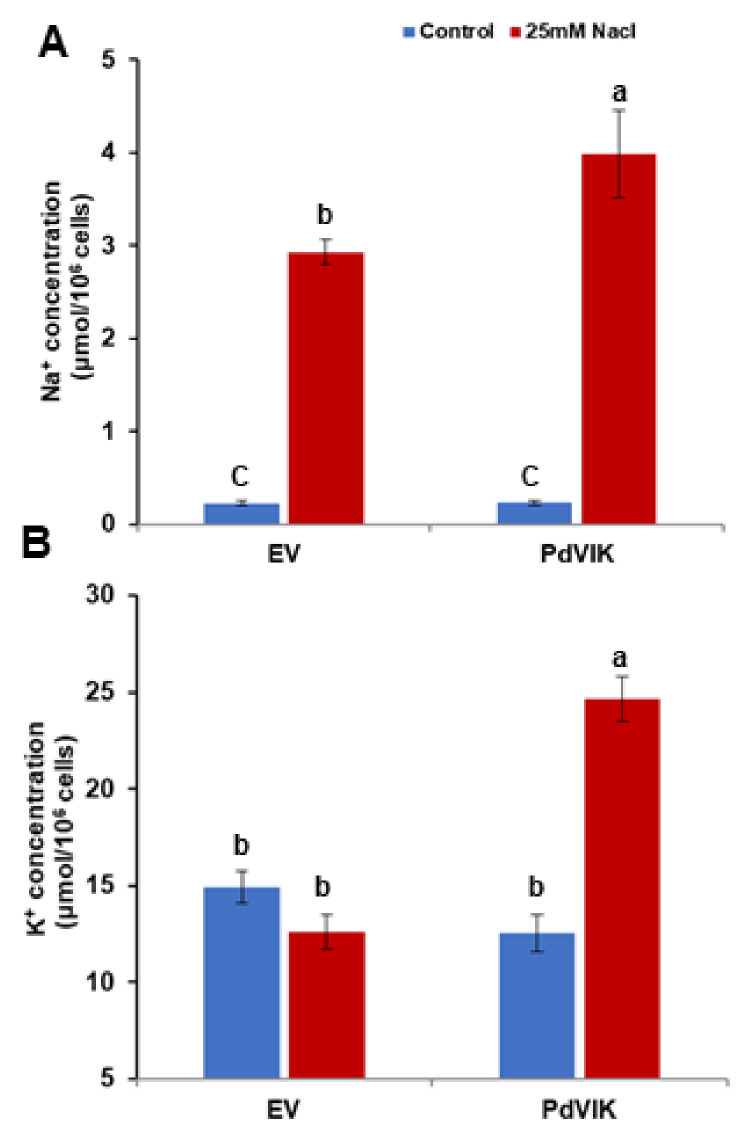
Intracellular Na^+^ (**A**) and K^+^ (**B**) concentrations in *PdVIK* and EV yeast cells grown on liquid synthetic medium (LSM) (control) and LSM supplemented with 25 mM NaCl. The bars represent the mean concentration ± SE of three independent biological replicates. The bars with different letters are significantly different at *p* < 0.05. The experiment was conducted three times.

Remarkably, although both EV and transgenic yeast grew at similar rates under NaCl stress, the Na^+^ levels were significantly (*p* < 0.05) higher in transgenic yeast compared to EV cells (Figure 5A), suggesting that the transgenic yeast cells are salt-tolerant despite accumulating higher Na^+^ levels. We also measured K^+^ accumulation patterns under salt stress in these cells. K^+^ accumulation did not differ between untreated and NaCl-treated conditions in EV cells. By contrast, the transgenic yeast accumulated significantly (*p* < 0.05) higher levels of K^+^ than the EV under salt stress (Figure 5B). These observations imply that the PdVIK expression in yeast is somehow promoting K^+^ accumulation under salt stress. 

**Figure 6:**
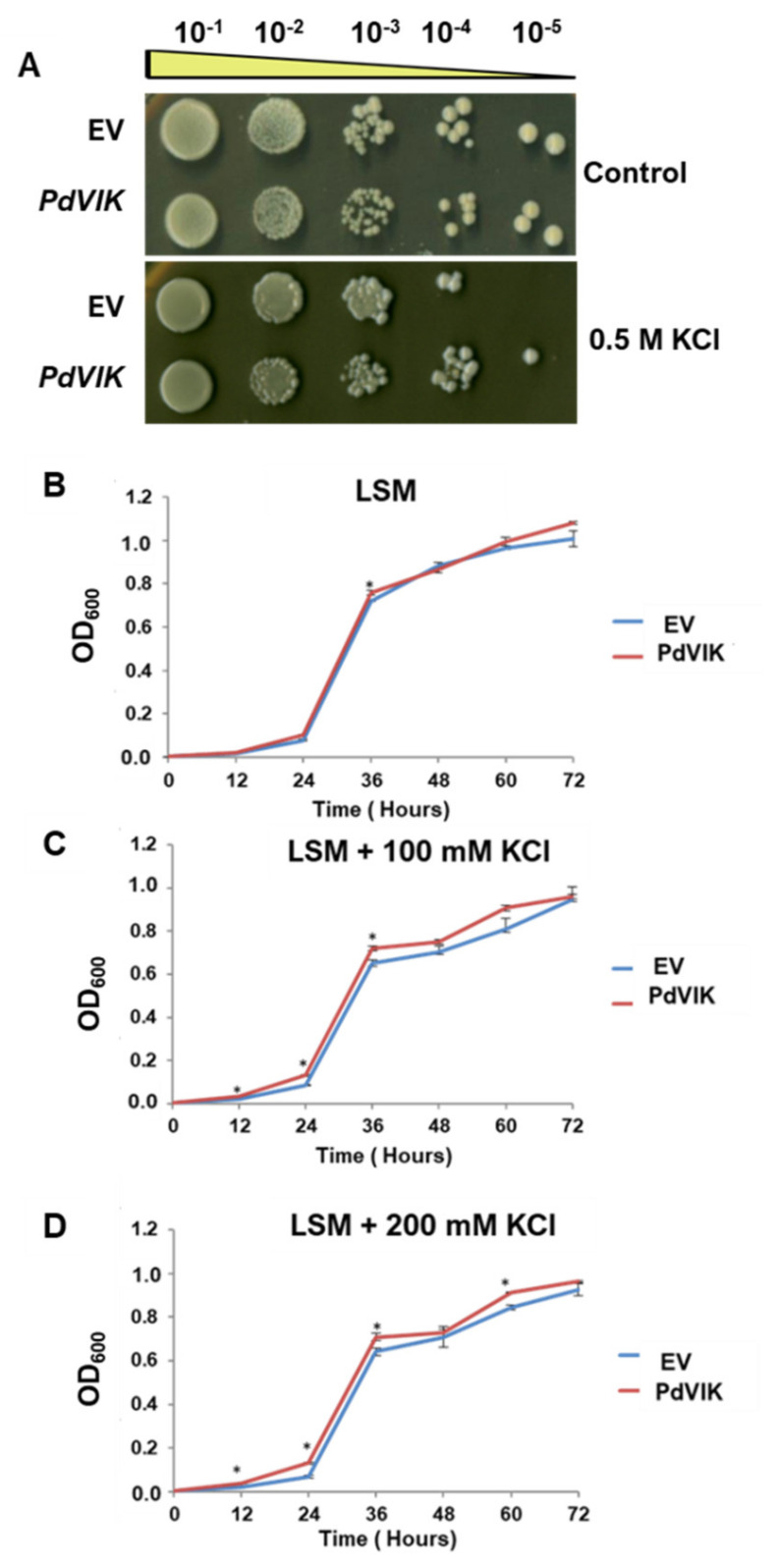
The growth response of transgenic *PdVIK* and EV yeast cells on SSM (Control) and SSM supplemented with 0.5 M KCl (**A**). The growth response of yeast cells cultured on liquid medium, LSM (control) (**B**), and on LSM supplemented with 100 mM KCl (**C**) or 200 mM KCl (**D**). Each optical density (OD) value represents the mean ± SE of three independent biological replicates and the statistical significance at *p* < 0.05 is indicated by an asterisk (*). This experiment was conducted three times.

Because PdVIK enhanced K^+^ accumulation specifically under salt stress conditions (Figure 5B), we were curious to assess whether the transgenic yeast would perform better under K^+^ enriched conditions. Therefore, the growth and K^+^ accumulation patterns were compared between PdVIK and EV yeast cells by culturing in a K^+^ rich solid and liquid media. Indeed, the PdVIK yeast cells exhibited better growth than the EV cells, on SMM supplemented with 0.5 M KCl (Figure 6A). Likewise, PdVIK yeast cells showed significantly (*p* < 0.05) increased growth rates compared to the EV yeast cells on LSM (Figure 6B), as well as on LSM supplemented with 100 and 200 mM KCl (Figure 6C,D).

### 3.4. The Transgenic Arabidopsis Seedlings Maintained Better Root Growth Under Stress Conditions 

**Figure 7:**
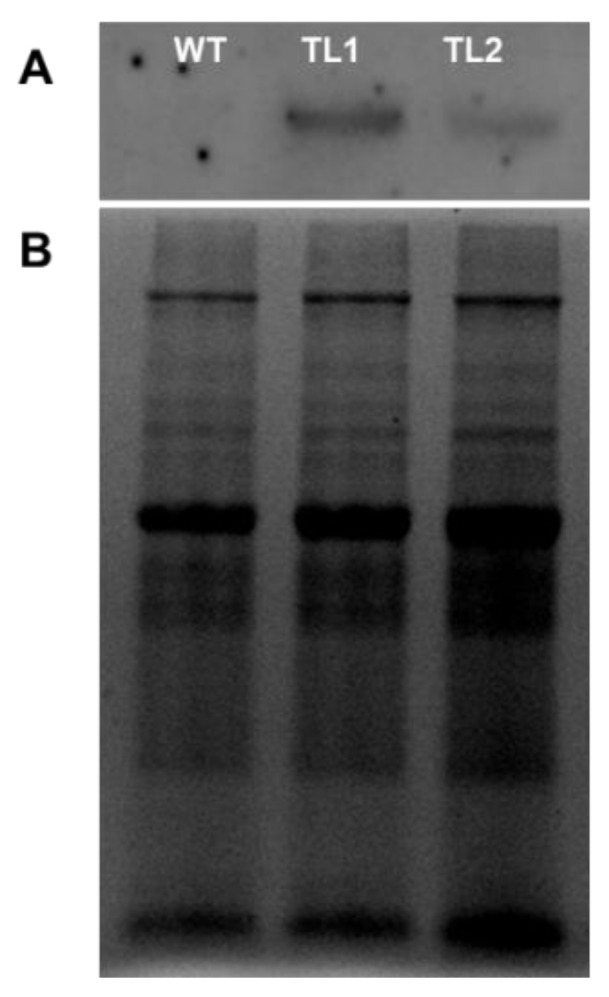
Generation and molecular characterization of the transgenic Arabidopsis lines. The immunoblot depicting the expression of PdVIK-myc in wild-type (WT) and two *PdVIK* transgenic lines (**A**). Total protein (100 µg), extracted from the WT and two independent homozygous transgenic Arabidopsis lines (TL1 and TL2), was loaded on a stain-free TGX polyacrylamide gel. The gel image was used as a loading control (**B**).

To ascertain the function of *PdVIK* in planta, two homozygous transgenic Arabidopsis lines (T3) were selected and used for morphological and physiological analyses. The *PdVIK* was fused in-frame with the Myc epitope to track the expression levels in transgenic lines. The presence of the transgene in the Arabidopsis lines was initially verified using PCR analysis (Figure S5A). Furthermore, using Myc-specific (epitope) antibodies, the accumulation level of the PdVIK protein in transgenic lines was determined (Figure 7A,B).

**Figure 8:**
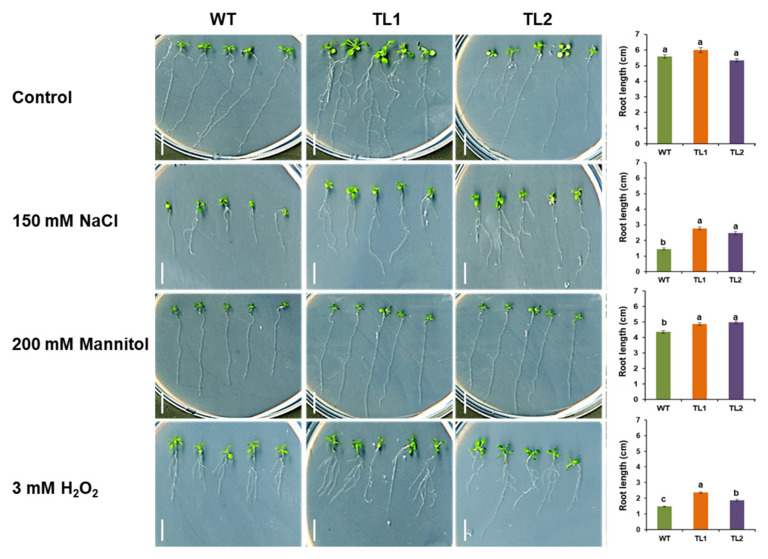
The effect of PdVIK on primary root length in response to different abiotic stresses. The root length was measured after 14 days of transfer to different stress conditions, such as the control, 150 mM NaCl, 200 mM mannitol, and 3mM H_2_O_2_. WT and two independent homozygous transgenic Arabidopsis lines (TL1 and TL2) were used in this analysis. Each genotype is presented in a column. The bars represent the mean root length ± SE of four independent replicates (an average length of the five plants (one replicate) was calculated and the calculated averages from the four replicates were averaged to obtain the mean root length). The bars with different letters are significantly different at *p* < 0.05. Scale bar =1 cm.

Although the *PdVIK* gene was induced by salt stress [28] it could also be important in drought stress responses because the osmotic and oxidative stresses are common to both salt and drought [51]. Therefore, we evaluated not only salt but also the drought tolerance potential of the *PdVIK* transgenic lines and compared them with WT plants. To assess the phenotypic effects of the transgene, the transgenic lines were grown along with WT, on MS medium and soil, and exposed to stress conditions. For MS medium assay, four-day-old seedlings grown on half-strength MS medium containing agar plates (control) were transferred to the MS-agar plates (control) or MS-agar plates supplemented with 150 mM NaCl (salt stress), 200 mM mannitol (osmotic stress), or 3mM H_2_O_2_ (oxidative stress). After 14 days of incubation, the root length was measured. Under control conditions (MS-agar alone), both WT and transgenic lines did not differ in their growth pattern, including root growth (Figure 8). However, under 150 mM NaCl, the transgenic lines had significantly (*p* < 0.05) longer primary roots and bigger leaves than the WT plants (Figure 8). Similarly, the transgenic lines had significantly (*p* < 0.05) longer roots than the WT seedlings exposed to 200 mM mannitol (Figure 8) or 3 mM H_2_O_2_ (Figure 8).

### 3.5. Soil Grown PdVIK Transgenic Lines Showed Modulated Responses to Salinity and Drought 

**Figure 9:**
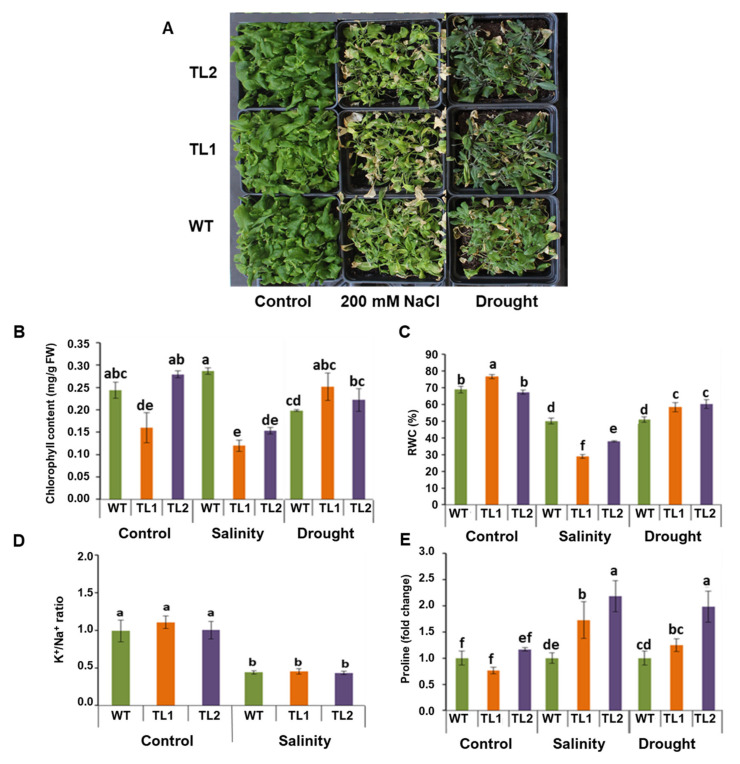
The soil-grown WT and two independent homozygous transgenic Arabidopsis lines (TL1 and TL2) exposed to salinity (200 mM NaCl) or drought (**A**). The effect of *PdVIK* overexpression on various physiological parameters in Arabidopsis subjected to salinity (200 mM) and drought treatments. The chlorophyll content (**B**), relative water content (**C**), K^+^/Na^+^ ratio (**D**), and proline concentration (**E**) of WT and two independent homozygous *PdVIK* transgenic Arabidopsis lines (TL1 and TL2), exposed to salinity and drought. The bars represent the mean value ± SE of three independent replicates. The bars with different letters are significantly different at *p* < 0.05.

Transgenic lines grown on soil were evaluated for the presence of morphological and physiological changes in response to drought and salinity stress (Figure 9A). After salt treatment, the EC of the control and treated soils were 0.63 ± 0.18 and 45.50 ± 1.40 dS/m, respectively (Supplementary Table S4). For the drought treatment, the soil moisture was found to be 0.20 ± 0.03 mm, while it was 9.12 ± 0.28 mm in the control soil (Supplementary Table S4). Different physiological parameters such as chlorophyll content, relative water content (RWC), K^+^/Na^+^ ratio, and proline accumulation were quantified in the WT and transgenic lines after salinity (200 mM) and drought treatments. Chlorophyll is the key pigment that reflects vigor and photosynthetic efficiency in plants. Interestingly, the total chlorophyll content was significantly (*p* < 0.05) reduced, even under control conditions, in the transgenic line (TL1) compared to the WT plants (Figure 9B). Under salinity, the chlorophyll content was significantly (*p* < 0.05) reduced in two transgenic lines compared to the WT plants. On the other hand, under drought, the chlorophyll levels were slightly increased in the transgenic lines compared to the WT (Figure 9B). Tolerance to drought depends largely on the plant’s ability to maintain better plant–water relations. One of the transgenic lines (TL1) had a significantly (*p* < 0.05) higher relative water content (RWC) under control conditions. However, under drought conditions, the two transgenic lines showed a significantly (*p* < 0.05) higher RWC than the WT (Figure 9C). However, under salinity, the two transgenic lines had a significantly (*p* < 0.05) lower RWC compared to the WT plants.

Since the K^+^/Na^+^ ratio is a critical factor determining ion homeostasis and salt tolerance in plants, K^+^ and Na^+^ concentrations were measured in plant tissues subjected to salt stress. While the K^+^/Na^+^ ratios were significantly (*p* < 0.05) reduced in response to salinity both in the transgenic lines and WT. The K^+^/Na^+^ ratios did not vary between transgenic lines and WT plants, either in the control treatment or under salt stress (Figure 9D). 

The abundances of proline in plants exposed to salt or drought are thought to be associated with stress tolerance. Therefore, proline levels were measured in the plant tissues. The proline levels differed between the transgenic lines under control conditions; TL2 had slightly more proline, while TL1 had slightly less than the WT plants. Under both salinity and drought treatments, the proline content was significantly (*p* < 0.05) increased in the transgenic lines, especially in TL2 (Figure 9E). While transgenic lines showed tolerance to drought stress, the results showed that overexpression of PdVIK did not help the transgenic Arabidopsis plants to recover after drought treatment.

## 4. Discussion

The relatively salt- and drought-tolerant nature of the date palm persuaded us to identify and functionally characterize its genes that respond to stress conditions. In this pursuit, our previous study has identified the *PdVIK* gene as one of the salt-stress-inducible genes in date palm [28]. This gene was previously annotated as *STK* (kinase), based on the amino acid sequence similarity to other kinases from other plant species, but its enzymatic activity is unknown. In this study, an enzymatic assay was performed to ascertain the function of this protein in vitro. In general, the protein kinases act on different substrates [52] depending on the complementation between the specificity determining residues (SDRs), which are shared between the two loops of the catalytic protein kinase domain and the substrate. Thus, the structural conformation of the binding will determine the availability of tyrosine, serine, or threonine residues for phosphorylation [53]. In general, MAPKKKs can phosphorylate serine/threonine/tyrosine residues [54]. However, PdVIK showed abundant tyrosine phosphorylation activity under the reaction conditions used in the present study (Figure 3), suggesting that the complementation between PdVIK subsites and MBP substrate side chains has positioned tyrosine residues in the vicinity of the active site of PdVIK. This part of the work provided evidence that PdVIK is a kinase through its phosphorylation activity. The determination of a kinase’s activity requires the use of various substrates, and this needs a comprehensive assessment of PdVIK activity assays. Future identification of in vivo substrates in the date palm can shed more light on the nature of the downstream target of PdVIK and its involvement in salt stress signal transduction.

Evidence for the direct involvement of PdVIK in salt tolerance could come from the observation that the transgenic yeast accumulated significantly more Na^+^ than the EV yeast, under salt stress, yet displayed better salt tolerance relative to the EV yeast (Figure 4 and Figure 5). Interestingly, the transgenic yeast also accumulated significantly greater amounts of K^+^ under salt stress than the EV yeast cells (Figure 5). The overall improved tolerance of PdVIK transgenic yeast could partly be due to enhanced K^+^ accumulation, which may contribute to osmotic adjustment under salt stress. Consistent with our findings, it has been previously shown that activation of the vacuolar Na^+^/H^+^ exchanger (NHX1) through phosphorylation by serine/threonine-protein kinase (SOS2), enhances Na^+^ pumping into the vacuole in yeast [16,17]. 

Overall, our results suggest that the overexpression of the kinase in yeast improves salt tolerance by enhanced K^+^ accumulation under salt stress. It has been previously shown that the overexpression of two homologous protein kinases (Hal4 and Hal5) in yeast cells activates K^+^ uptake by modulating Trk1 and Trk2 K^+^ transporters [55]. Potassium uptake reduces the electrical driving force and, hence, decreases the uptake of toxic cations, such as lithium [56].

Consistent with the results of our studies in yeast, PdVIK overexpression in Arabidopsis also enhanced salt, osmotic/drought, and oxidative stress tolerance (Figure 8; Figure 9). Under drought, the transgenic Arabidopsis lines showed better RWC than the WT plants (Figure 9C). However, under salt stress, a trend of enhanced proline accumulation in the transgenic plants stress compared to WT plants, may indicate that overexpression of *PdVIK* contributes to osmotic adjustment and decreases oxidative stress levels. Overexpression of kinases enhanced tolerance to various abiotic stresses in other plant species. For example, overexpression of *Gossypium barbadense* receptor-like kinase (*GbRLK*) in Arabidopsis improved tolerance against drought and salinity stress, by reducing water loss from the plants; this was achieved by control of an ABA-dependent signaling pathway and the expression of antioxidant related genes [27,46]. Likewise, overexpression of *Pohlia nutans* leucine-rich repeat receptor-like kinase 27 (LRR-RLK27), in Arabidopsis, enhanced oxidative stress and salinity tolerance [57], and the overexpression of wheat sucrose non-fermenting1-related protein kinase 2 (SnRK2.8), in Arabidopsis, improved the salinity, drought, and low-temperature tolerance of transgenic lines [26].

It was shown recently that a potato VIK is targeted by the *Phytophthora infestans* RXLR-type effector protein (Pi17316) in promoting the disease caused by this pathogen, implicating that VIK also has a role in biotic stress response [49]. The C1 subclass of MAPKKKs (VIKs) possesses an N-terminal ankyrin repeat domain (Figure 2), which is predicted to interact with a range of different protein substrates, suggesting that the VIKs are not exclusively associated with the MAPK cascades in plants (MAPK Group, 2002) [49]. Therefore, it is possible that PdVIK is a component of biotic and abiotic and other signal transduction pathways in the date palm.

The importance of the MAPK cascade in plant abiotic stress response is well characterized [58]. It is also well known that the MAPKKKs can phosphorylate several MAPKKs, and, consequently, activate multiple downstream targets, which in turn, play essential roles in adaptation to stress conditions. Besides, the upstream component activates the MAPKKK in response to the environmental stimuli [59]. Identifying those upstream and downstream components is an important goal for the future.

## 5. Conclusions

The present study reveals a role for *PdVIK* in different abiotic stress responses. Morphologically, *PdVIK* overexpression resulted in better root growth compared to wild-type under stress conditions. Physiologically, *PdVIK* can modulate the ionic uptake process in yeast cells and promote proline accumulation in stress-exposed transgenic plants. Despite the fact that *PdVIK* showed some positive effect on the salt and drought treated transgenic Arabidopsis plants, its effect was only subtle. Nevertheless, the results shown in this study have demonstrated sufficient evidence to support the idea that *PdVIK* gene plays an important role under stress conditions in the date palm.

## Supplementary Materials

The following are available online at https://www.mdpi.com/2073-4425/11/5/568/s1, Table S1: Oligonucleotides used in this study; Table S2: In silico identification of the protein kinase gene family coded by the date palm genome; Table S3: Abiotic stress-responsive kinases in the date palm; Table S4: Moisture, temperature, and electrical conductivity (EC) of the soil of the pots after treatments. Values represent the mean ± SD of three independent replicates; Figure S1. Phylogenetic tree of the date palm, Arabidopsis, and rice protein kinases constructed using the Neighbor Joining (NJ) algorithm. Numbers on the nodes indicate the percentage of 1000 replicates bootstrap analysis. Phylogenetic analysis revealed that PdVIK (blue font) was closely related to Arabidopsis (AtVIK) and potato (StVIK); Figure S2. Multiple sequence alignment of deduced amino acid sequences of PdVIK and other VIK proteins from Arabidopsis (AtVIK), potato (StVIK), and rice (OsVIK). PdVIK protein conserved regions (dark grey-colored highlights) that are shared with other kinases. The red box highlights the ankyrin repeat domain, which is predicted to interact with a range of different protein substrates. The blue box highlights the kinase catalytic (PKc), which consists of a polypeptide substrate binding site, the ATP binding site, and the protein kinases active site (ACT site); Figure S3. Motif structure of 53 abiotic stress-responsive kinase proteins of the date palm using multiple expectation maximization for motif elicitation (MEME) platform. MEME parameters were set to detect a maximum of 40 motifs with a width coverage of 6 to 50 amino acid residues. PdVIK protein shared seven motifs with the integrin-linked protein kinase (ILK) and serine/threonine PK2/PK19 kinases. The asterisk indicates a unique motif only shared between PdVIK and PdILK1, which may have an essential role in these two proteins; Figure S4. The abundance and the distribution of cis-acting regulatory elements within PdVIK putative promoter region. The abundance of the regulatory elements involved in abiotic stress compared with other general regulatory elements within PdVIK putative promoter region (2000 bp), computationally analyzed using PlantPAN 3.0 database (A). The distribution of cis-acting regulatory elements with their binding sites among the putative proximal region of PdVIK promoter (950 bp) analyzed by Plant CARE database. Most of these elements are related to abiotic stress responses, such as CAAT, DRE, GT1 motif, MYB, MYC, and AP2 (B); Figure S5. PCR-based genotyping of PdVIK transgenic Arabidopsis plants using the 35S forward promoter and gene-specific reverse primers (VIKR) (upper), as well as gene-specific forward (VIKF) and OCS terminator reverse primers (lower) (A). The immunoblot image shows the expression of PdVIK-myc in WT and two PdVIK transgenic lines (B). The TGX polyacrylamide gel image shows total protein samples from two homozygous PdVIK transgenic Arabidopsis lines (TL1 and TL2) and WT, used for Western blot analysis (C).
