# Supplementary material for: Molecular Characterization of a Date Palm Vascular Highway 1-Interacting Kinase (PdVIK) under Abiotic Stresses
Source: Genes (Basel). 2020 May 19;11(5):568. doi: 10.3390/genes11050568 (PMC7288329; doi:10.3390/genes11050568)
Supplement: Supplementary file 1 [file genes-11-00568-s001.zip › supplementary/Supplemetary figures (29-4-20).pdf]

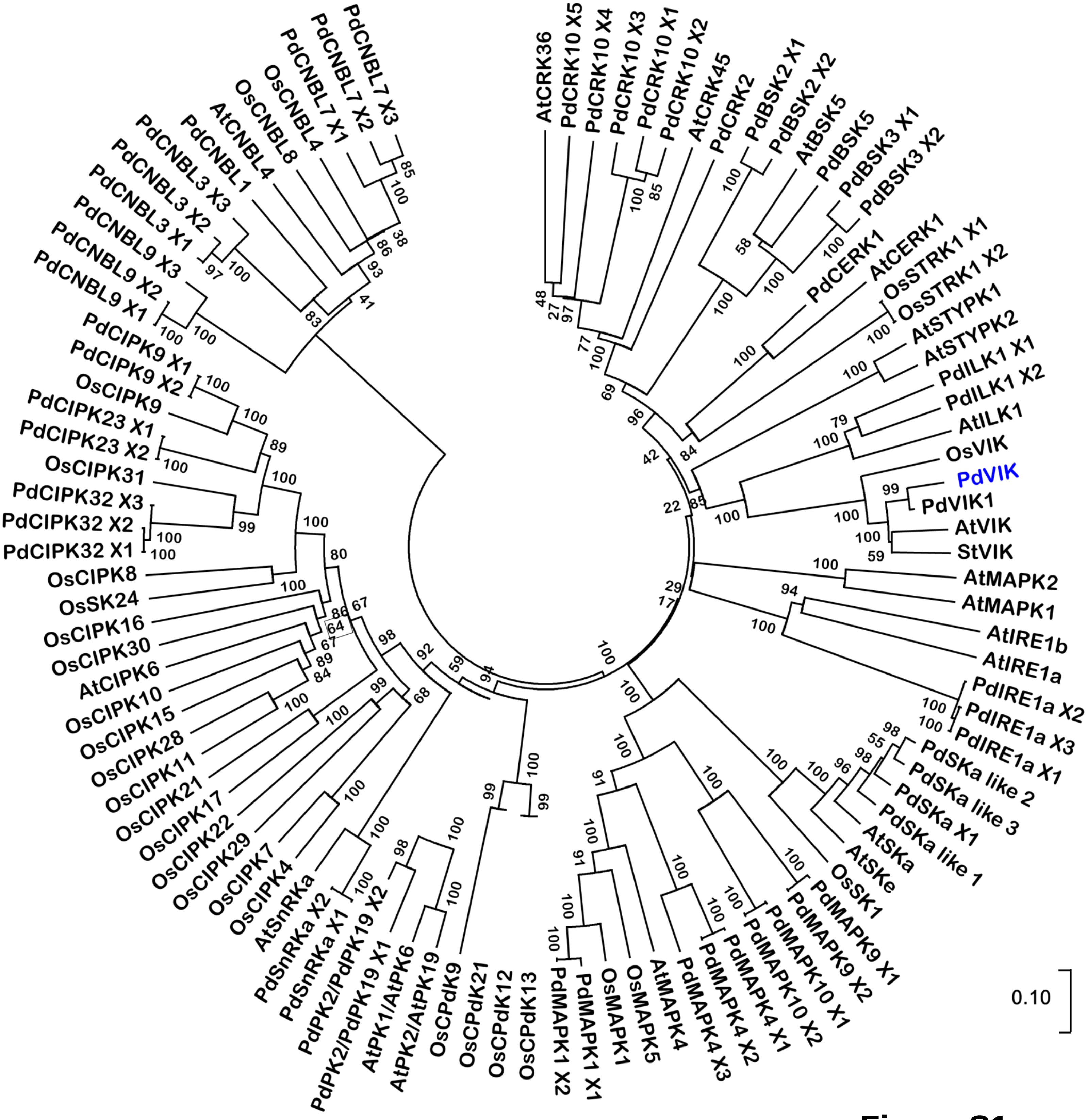
PdCNBL7 X3
PdCNBL7 X2
PdCNBL7 X1
OsCNBL4
OsCNBL8
AtCNBL4
PdCNBL1
PdCNBL3 X3
PdCNBL3 X2
PdCNBL3 X1
PdCNBL9 X3
PdCNBL9 X2
PdCNBL9 X1
PdCIPK9 X1
PdCIPK9 X2
OsCIPK9
PdCIPK23 X1
PdCIPK23 X2
OsCIPK31
PdCIPK32 X3
PdCIPK32 X2
PdCIPK32 X1
OsCIPK8
OsSK24
OsCIPK16
OsCIPK30
AtCIPK6
OsCIPK10
OsCIPK15
OsCIPK28
OsCIPK11
OsCIPK21
OsCIPK17
OsCIPK22
OsCIPK29
OsCIPK7
OsCIPK4
OsSnRKa
AtSnRKa
PdSnRKa X2
PdSnRKa X1
PdPK2/PdPK19 X2
PdPK2/PdPK19 X1
AtPK1/AtPK6
AtPK2/AtPK19
OsCPdK9
OsCPdK21
OsCPdK12
OsCPdK13
PdMAPK1 X2
PdMAPK1 X1
OsMAPK1
OsMAPK5
AtMAPK4
PdMAPK4 X3
PdMAPK4 X2
PdMAPK4 X1
PdMAPK10 X2
PdMAPK10 X1
PdMAPK9 X2
PdMAPK9 X1
OsSK1
AtSKe
AtSKa
PdSKa like 1
PdSKa X1
PdSKa like 3
PdSKa like 2
PdIRE1a X1
PdIRE1a X3
PdIRE1a X2
AtIRE1a
AtIRE1b
AtMAPK1
AtMAPK2
StVIK
AtVIK
PdVIK1
PdVIK
OsVIK
AtILK1
PdILK1 X2
PdILK1 X1
AtSTYPK2
AtSTYPK1
OsSTRK1 X2
OsSTRK1 X1
AtCERK1
PdCERK1
PdBSK3 X2
PdBSK3 X1
PdBSK5
AtBSK5
PdBSK2 X2
PdBSK2 X1
PdCRK2
AtCRK45
PdCRK10 X2
PdCRK10 X1
PdCRK10 X3
PdCRK10 X4
PdCRK10 X5
AtCRK36
0.10

Figure S1

```
PdVIK    1   MTPRKLEERASHRSDLLDRRREMSGGGGEEASGGSEGTSSGTSSAASTEKQREKARVSRT   60
AtVIK    1   ----------------------MSSDSPAAGDGGEQAAAGTSVPSPSYDKQKEKARVSRT   38
StVIK    1   -------------------------MSTSEGSSGSSAAAAVEVPVVSTEKKKEKSRVSKT   35
OsVIK    1   MSGAE-EEAAAARGGG---SGSESGGSGGGGRGGGGGGAGSSGSSGARRRFDDKGLVART   56
                                     .   . .*  . :.        :  :  :*.  *::*

PdVIK   61   SLILWHTHQNDASAVRKLLEEDPGLVNARDYDNRTPLHVASIHGWIDVAKCLLEYGADVN  120
AtVIK   39   SLILWHAHQNDAAAVRKLLEEDPTLVHARDYDKRTPLHVASLHGWIDVVKCLLEFGADVN   98
StVIK   36   SQILWHAHHNDAAAVRKLLEEDRTLVQARDYDNRTPLHVAALHGWIDVAKCLIDYGADVN   95
OsVIK   57   SLILWHTHQNDAGAVRKLLEEDAALVNARDYDSRTPLHVAALHGWHDVAECLIANGADVN  116
             * ****:*:***.*********  **:*****.*******::***  **.:**:   *****

PdVIK  121   AQDRWKNTPLADAEGAKKHNMIELLKSYGGLSFGQNGSHFEPKPVPPPLPNKCDWEIEPS  180
AtVIK   99   AQDRWKNTPLADAEGARKQKMIELLKSHGGLSYGQNGSHFEPKPVPPPIPKKCDWEIEPA  150
StVIK   96   AQDRWRNTPLADAEGAKKSAMIELLKTYGGLSYGQNGSHFEPRPVAPPLPKKCDWEIDPT  155
OsVIK  117   AQDRWQNTPLADAEGAKRHAMIELLKEHGGLTYGKTGSHFEPKTIPPPLTNKADWEINPL  176
             *****:**********::  ****** :***::*:.******: : **:  :*.****:*

PdVIK  181   ELDFSASTIIGKGSFGEILKAYWRGTPVAVKRILPSLSEDRLVIQDFRHEVNLLVKLRHP  240
AtVIK  159   ELDFSNAAMIGKGSFGEIVKAYWRGTPVAVKRILPSLSDDRLVIQDFRHEVDLLVKLRHP  218
StVIK  156   ELDFSNSAIIGKGSFGEILKACWRGTPVAVKRILPNLSDDRLVIQDFRHEVNLLVKLRHP  215
OsVIK  177   ELDFSKAVIIGKGSFGEILKANWRGTPIAVKRILPSLSDDRLVIQDFKHEVNLLIKLRHP  236
             ***** :.:*********:** *****:*******.**:********:***:**:*****

PdVIK  241   NIVQFLGAVTERKPLMLITEYLRGGDLHQFLKEKGSLTPLTAVNFALDIARGMAYLHSEP  300
AtVIK  219   NIVQFLGAVTERKPLMLITEYLRGGDLHQYLKEKGGLTPTTAVNFALDIARGMTYLHNEP  278
StVIK  216   NIVQFLGAVTEKKPLMLITEYLRGGDLHQHLKEKGALTPSTAINFAMDIARGMTYLHNEP  275
OsVIK  237   NIVQFLGAVTETKPLMLVTEFLRGGDLHQYLKEKGALAPATAVNFALDIARGMAYLHNEP  296
             *********** *****:**:********.*****.*:* **:***:******:***.**

PdVIK  301   NVIIHRDLKPRNVLLVNSNADHLKVGDFGLSKLIRVQNSHDVYKMTGETGSYRYMAPEVF  360
AtVIK  279   NVIIHRDLKPRNVLLVNSSADHLKVGDFGLSKLIKVQNSHDVYKMTGETGSYRYMAPEVF  338
StVIK  276   NVIIHRDLKPRNVLLVNSNADHLKVGDFGLSKLIRVQNSHDVYKMTGETGSYRYMAPEVF  335
OsVIK  297   NVVIHRDLKPRNILLVNSAANHLKVGDFGLSKIIKAQHANDVYKMTGETGSYRYMAPEVF  356
             **:*********:*****  *:**********:*:.*:::********************

PdVIK  361   KHRKYDKKVDVFSFAMILYEMLEGDPPFSNYEAYEGAKYAADGHRPTFRAKGYVQELKEL  420
AtVIK  339   KHRRYDKKVDVFSFAMILYEMLEGEPPFANHEPYEAAKHVSDGHRPTFRSKGCTPDLREL  398
StVIK  336   KHRKYDKKVDVFSFAMILYEMLEGDPPLSHYEPYEAAKYVAEGHRPMFRAKGFTPELKEL  395
OsVIK  357   KHRKYDKKVDVFSFAMILYEMLEGDPPFSNYEPYEAAKYVGEGHRPPFRSKGFTNELKEL  416
             ***:********************:**::::* **.**:..:**** **:**  .  :*:**

PdVIK  421   TSQCWSADMNQRPSFLEILKRLEKIKEILP-QEHHWHIL--                    458
AtVIK  399   IVKCWDADMNQRPSFLDILKRLEKIKETLP-SDHHWGLFTS                    438
StVIK  396   VEECWAPDMNKRPSFLDILKRLEKIKDALP-SEHHWHLFTS                    435
OsVIK  416   IELCWSGDIHLRPSFLEILKRLEKIKEYLAAHDHHWHLFSQ                    457
                **  *::  *****:*********: *    :***  ::
```

Figure S2

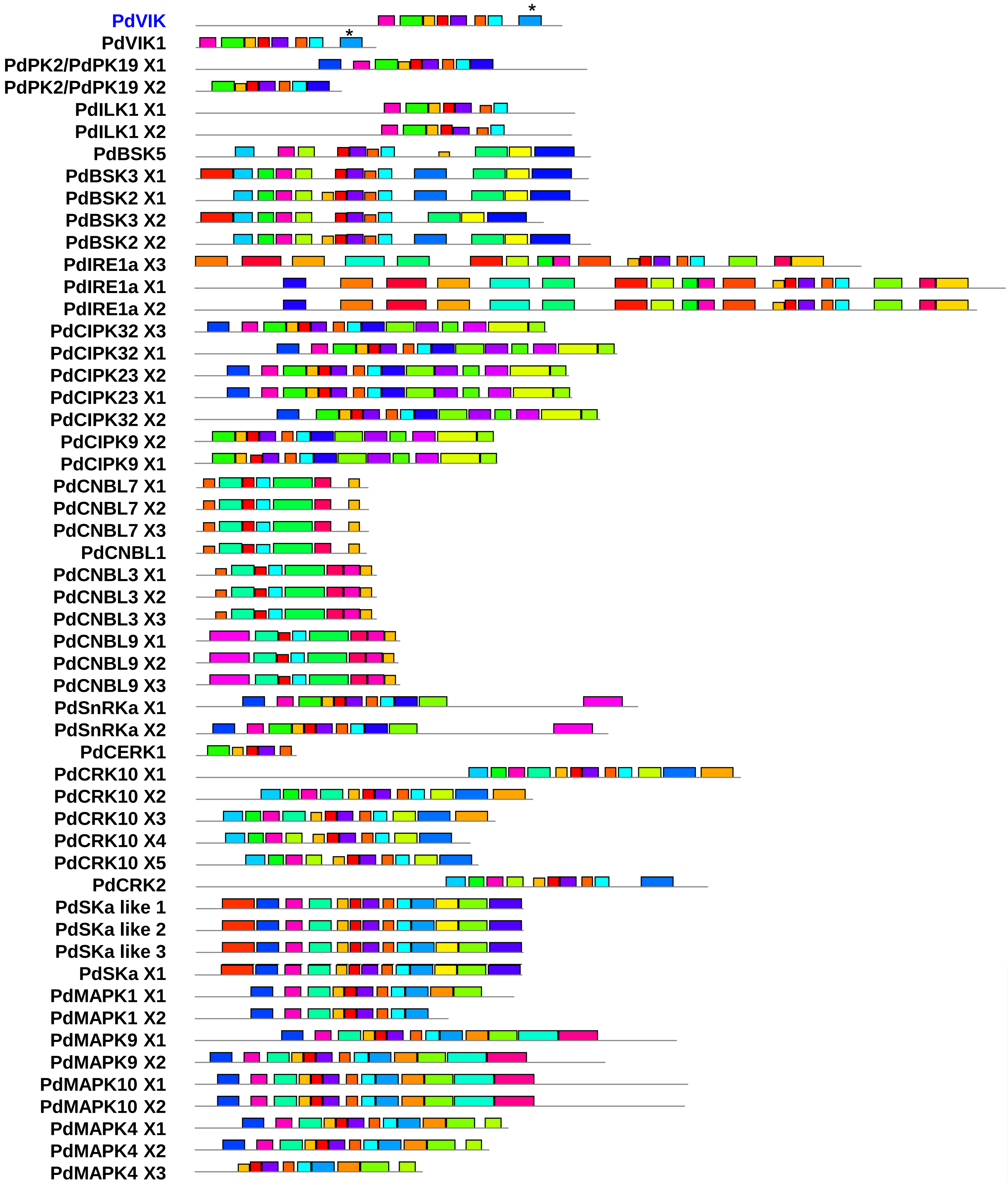
PdVIK
PdVIK1
PdPK2/PdPK19 X1
PdPK2/PdPK19 X2
PdILK1 X1
PdILK1 X2
PdBSK5
PdBSK3 X1
PdBSK2 X1
PdBSK3 X2
PdBSK2 X2
PdIRE1a X3
PdIRE1a X1
PdIRE1a X2
PdCIPK32 X3
PdCIPK32 X1
PdCIPK23 X2
PdCIPK23 X1
PdCIPK32 X2
PdCIPK9 X2
PdCIPK9 X1
PdCNBL7 X1
PdCNBL7 X2
PdCNBL7 X3
PdCNBL1
PdCNBL3 X1
PdCNBL3 X2
PdCNBL3 X3
PdCNBL9 X1
PdCNBL9 X2
PdCNBL9 X3
PdSnRKa X1
PdSnRKa X2
PdCERK1
PdCRK10 X1
PdCRK10 X2
PdCRK10 X3
PdCRK10 X4
PdCRK10 X5
PdCRK2
PdSKa like 1
PdSKa like 2
PdSKa like 3
PdSKa X1
PdMAPK1 X1
PdMAPK1 X2
PdMAPK9 X1
PdMAPK9 X2
PdMAPK10 X1
PdMAPK10 X2
PdMAPK4 X1
PdMAPK4 X2
PdMAPK4 X3
*
*

Figure S3

A

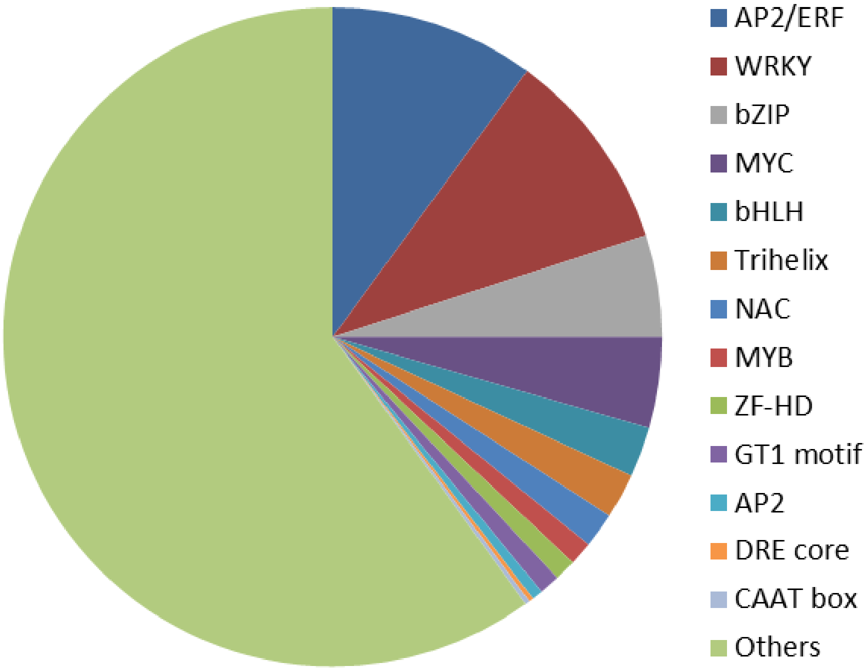

B

```
+ TGTTGACTCT TTTGGATGTA CTCATACTAG ATATGTATGA TTTGTCTATC TTAGCAAAAT AAAAATAAAA
- ACAACTGAGA AAACCTACAT GAGTATGATC TATACATACT AAACAGATAG AATCGTTTTA TTTTTATTTT

+ ATAATGAATT TTTATTAGAA TAATTTTTAA TCAATTTATT AGCTAAACAT ATTTTAGAGA ATCACTTAAT
- TATTACTTAA AAATAATCTT ATTAAAAATT AGTTAAATAA TCGATTTGTA TAAAATCTCT TAGTGAATTA

+ CAAAAAAATA AGTCAATAAT TTTCAAATAA ATTAAGCATT TTCACAAAAA AATGCAGGTT CATTCTAAAC
- GTTTTTTTAT TCAGTTATTA AAAGTTTATT TAATTCGTAA AAGTGTTTTT TTACGTCCAA GTAAGATTTG

+ TTTTATTAGT CTAATTTTGA AGCACTAGAG TCTTGATGCT AGACAAGATC AAATCAAGGG TAGCTAATGC
- AAAATAATCA GATTAAAACT TCGTGATCTC AGAACTACGA TCTGTTCTAG TTTAGTTCCC ATCGATTACG

+ AAAAGTTAAT AATGAAAGAG CGAATTGATC CGATGAGATC AGCTCAAGAA AGAAAATAAT AACCAGATTG
- TTTTCAATTA TTACTTTCTC GCTTAACTAG GCTACTCTAG TCGAGTTCTT TCTTTTATTA TTGGTCTAAC

+ TGACAAAATT CTTATCAAAT GTAGTGGATA CCATGTCTTT TCTTCCTTTA TACCATATGA AATATCCACA
- ACTGTTTTAA GAATAGTTTA CATCACCTAT GGTACAGAAA AGAAGGAAAT ATGGTATACT TTATAGGTGT

+ TGTAATAAAA TTTGGACAAT CCCTATATTT TTTTCTCTTT ACTCACCCAA GACCCAACTT GTCTTTTTAT
- ACATTATTTT AAACCTGTTA GGGATATAAA AAAAGAGAAA TGAGTGGGTT CTGGGTTGAA CAGAAAAATA

+ TTTTTTTTCC GGAACATACA ATACATTTTC AAATGCAACA TTATGTGTAT TATTTACCAT ATTTACTACA
- AAAAAAAAGG CCTTGTATGT TATGTAAAAG TTTACGTTGT AATACACATA ATAAATGGTA TAAATGATGT

+ TTAGTCTGAT CAGAAGTCTA ACCTGCATTA TGTTTCGGTA TGCAACGTAT TTTATTCAAT CTACAAAAAC
- AATCAGACTA GTCTTCAGAT TGGACGTAAT ACAAAGCCAT ACGTTGCATA AAATAAGTTA GATGTTTTTG

+ CAGAAAGTTA AACGGAATAT ATTTGTGGAC TCCTACGTCA CAAGGCGTTT GCCGACAACT TACCGGGACC
- GTCTTTCAAT TTGCCTTATA TAAACACCTG AGGATGCAGT GTTCCGCAAA CGGCTGTTGA ATGGCCCTGG

+ CACCCCCTCT CCGTCCTCCC AAGCCGTAGC AAGAGCGAGT CAGAACTCCA AAACAAACCT TGAGCACGAG
- GTGGGGGAGA GGCAGGAGGG TTCGGCATCG TTCTCGCTCA GTCTTGAGGT TTTGTTTGGA ACTCGTGCTC

+ GACTGGATGG CGGATCTTGA TCCGCCGACC AGGTCGACTC GGCCGACTCC CGAGGCGGCG CGAGACGCCG
- CTGACCTACC GCCTAGAACT AGGCGGCTGG TCCAGCTGAG CCGGCTGAGG GCTCCGCCGC GCTCTGCGGC

+ ACCATTAACC GAACCGAAGC AAAGGCAAAA GATATTTTTC TATCTTCTTG GAGGGCATCT TCGAGGCAAA
- TGGTAATTGG CTTGGCTTCG TTTCCGTTTT CTATAAAAAG ATAGAAGAAC CTCCCGTAGA AGCTCCGTTT

+ ATAAAAACAG TAGAGGGTTG CTCCCTGCGG AGCCTCAGC
- TATTTTTGTC ATCTCCCAAC GAGGGACGCC TCGGAGTCG
```

- CAAT box
- DRE Core
- GT1 motif
- MYB
- MYC
- AP2

Figure S4

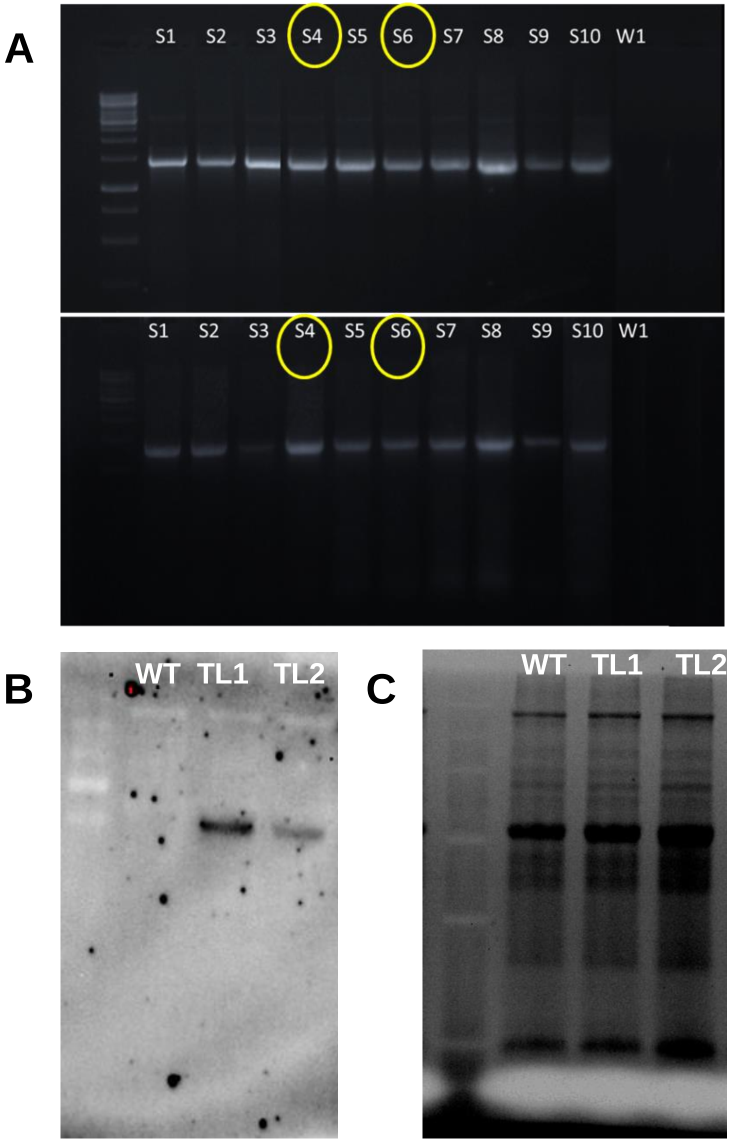
A
S1
S2
S3
S4
S5
S6
S7
S8
S9
S10
W1
S1
S2
S3
S4
S5
S6
S7
S8
S9
S10
W1
B
WT
TL1
TL2
C
WT
TL1
TL2

Figure S5
